# Online Health Information-Seeking Behavior and Confidence in Filling Out Online Forms Among Latinos: A Cross-Sectional Analysis of the California Health Interview Survey, 2011-2012

**DOI:** 10.2196/jmir.5065

**Published:** 2016-07-04

**Authors:** Mariaelena Gonzalez, Ashley Sanders-Jackson, Jason Emory

**Affiliations:** ^1^ Public Health University of California, Merced Merced, CA United States; ^2^ Health Sciences Research Institute University of California, Merced Merced, CA United States; ^3^ Department of Advertising and PR School of Communication Arts and Sciences Michigan State University East Lansing, MI United States; ^4^ Department of Psychology & Child Development California State University, Stanislaus Turlock, CA United States

**Keywords:** communications media, social media, health information-seeking behavior, online forms, Hispanic Americans

## Abstract

**Background:**

Health information is increasingly being disseminated online, but there is a knowledge gap between Latinos and non-Hispanic whites, particularly those whose English language proficiency is poor, in terms both of online health information-seeking behavior and computer literacy skills. This knowledge gap may also exist between US- and foreign-born Latinos.

**Objective:**

The specific aim of this study was to examine Internet use, online health information-seeking behavior, and confidence in filling out online forms among Latinos, particularly as it relates to health-risk behaviors. We then stratified our sample by nativity.

**Methods:**

We used the adult population file of the 2011-2012 California Health Interview Survey, analyzing Internet use, online health information-seeking behavior, and confidence in filling out online forms using binary logistic regression among Latinos and whites (N=27,289), Latinos (n=9506), and Latinos who use the Internet (n=6037).

**Results:**

Foreign-born Latinos (OR 0.71, 95% CI 0.58-0.88, *P*=.002) have lower odds of engaging in online health information-seeking behavior, and higher odds (OR 2.90, 95% CI 2.07-4.06, *P*<.001) of reporting a lack of confidence in filling out online forms compared to US-born Latinos. Correlates of online health information-seeking behavior and form confidence varied by nativity.

**Conclusions:**

Latinos, particularly foreign-born individuals, are at an increased risk of being left behind as the move to increase online content delivery and care expands. As online health information dissemination and online health portals become more popular, the impact of these sites on Latino gaps in coverage and care should be considered.

## Introduction

Health information and marketing is increasingly being disseminated and collected via the Internet [1]; therefore, understanding who is and is not engaging in online health information-seeking behavior is crucial to improving health. Health information-seeking behavior is the third most popular use of the Internet among adults [2,3]. The use of online and mobile interventions shows promising results as a way to implement large-scale behavioral changes [4]. As a result, tracking online health information-seeking behavior is particularly important because online health information-seeking behavior is associated with risk behaviors in the general population [5]. However, this association between online health information-seeking behavior and Internet use varies by behavior. Decreased fruit and vegetable consumption are associated with decreased use of the Internet for health information-seeking behavior, whereas others, such as smoking, are associated with increased online health information-seeking behavior in the general US population [5]. Additionally, it is important to understand competencies in performance of online tasks—such as the ability to fill out online forms—because insurers, hospitals, and other government programs are using online forms and online apps as ways of gathering information and communicating with their clients [6].

There are significant geographic, social, economic, and racial/ethnic disparities in online health information-seeking behavior [7,8]. In particular, although more than 75% of Latinos use the Internet or email at least occasionally [9], Latinos are less likely than other groups to go online for health information and to use online health services such as Internet portals [3,10-12]. Further, differences in English language literacy, eHealth literacy, and background knowledge may also affect how much benefit people receive from health-related information online [13-15]. We define eHealth literacy as “the ability to seek, find, understand, and appraise health information from electronic sources and apply the knowledge gained to addressing or solving a health problem” [16]. Although computer literacy is part of eHealth literacy, it is a fairly complex construct that allows scholars to understand how existing disparities can be exacerbated be electronic tools due to the ability of the existing system to reinforce structural inequalities [14].

Although English language proficiency has been increasing among Latinos overall [17], there continues to be disparities in eHealth literacy and health-related background knowledge. For example, Latinos are less likely than other groups to have high computer use efficacy and they have a lower activation rate for online health systems than whites [18,19]. In addition, in a study of parents in Florida, Latino parents had less success accessing an eHealth portal for help with their special needs children [4]. Latinos with poorer English language proficiency are less likely to seek health information online [15] and older Latinos with poorer English tend to have lower eHealth literacy [5]. Further, Latino women are less likely to seek health information online than white women [10], which may lead to online health information knowledge gaps. For example, Latinos typically have lower levels of cancer-related health information than whites do [20-22].

When examining Latinos, it is important to consider that there exists significant differences between US- and foreign-born Hispanics in the United States. Latinos born in the United States are generally younger and have higher socioeconomic status (as measured by education and income levels) than non-US-born Latinos [23,24]. With each successive generation born in the United States, English language dominance increases as Spanish usage declines. English language proficiency and nativity are also significant when considering Hispanic Internet usage in general [17,23]. A Pew survey shows that half of all American Hispanic Internet users are US-born and the majority of American Hispanic Internet users are either English dominant or bilingual [9].

Although some recent studies have been done on online health information-seeking behavior among Latinos to determine the profile of Latino online health information-seeking behavior, the majority of these studies have been small or limited to particular localities (ie, boroughs of New York or Puerto Rico) [11,25-28]. One study examined a national sample of Latinos [29]. We found no studies on use of the Internet for health information-seeking behavior by Latinos who engage in health-risk behavior. Furthermore, we found no study that stratifies Latinos by nativity in order to understand how the correlates of online health information-seeking behavior differs between foreign- and US-born Latinos. Understanding how online health information-seeking behavior by Latinos varies by nativity is important because the use of media for health information and prevalent modes of media use among Latinos differs according to demographic characteristics [29,30].

California is the most populous US state; in 2015, 38% of its population was Hispanic or Latino as compared to 17.4% in the US population overall [31]. Due to the high percentage of Hispanics in California, research related to Hispanic or Latino individuals often sample from there specifically [32-34]. As a result, understanding Internet access and online health information-seeking behavior of Latinos in California will help to predict national trends. When studying Latinos in California, it is particularly important to examine US- versus foreign-born Latinos because there is a significant demographic divide between Latinos in the United States and in California specifically; in 2011, the average age of US-born Latinos in California was 18 years and 72% of this population were younger than age 30 [35]. This mirrors the demographic shift that the Hispanic population in the United States has been undergoing over the last three decades. As a result, although Hispanics are still perceived as an immigrant population, in 2013, 64.8% of all US Hispanics were born in the United States [36].

The purpose of this study is to examine Internet use, online health information-seeking behavior, and confidence in filling out online forms (form confidence) among Latinos in California, particularly as it relates to health-risk behaviors. This study (1) examines whether or not disparities between Internet use, online health information-seeking behavior, and lack of confidence in filling out online forms varies between non-Hispanic whites and Latinos, (2) then analyzes Internet use and online health information-seeking behavior and lack of confidence in filling out online forms among Latinos, and (3) examines the correlates of health information-seeking behavior and confidence in filling out online forms among US- and foreign-born Latinos, stratifying by nativity.

## Methods

Data came from the adult population file of the 2011-2012 California Health Interview Survey (CHIS), which is a representative sample of noninstitutionalized California state adult population aged 18 and older [37]. We analyzed Latinos and non-Hispanic whites (N=27,289), all Latinos (n=9506), and the subsample of Latinos who reported using the Internet in the last 12 months (n=6037). Additionally we stratified our analyses of Latinos by nativity.

### Primary Outcomes

Our three primary outcomes of interest were (1) ever used the Internet, defined as having ever used the Internet including email and social media sites; (2) health information-seeking behavior in the last 12 months, defined as seeking health or medical information online, including information about disease symptoms, diet or nutrition, physical activity, health care providers, and health insurance plans; and (3) lack of confidence in filling out an online application on their own (not at all confident or not too confident=1, somewhat confident or very confident=0).

### Sociodemographic and Health Risk Variables

We controlled for age (18-24, 25-34, 35-44, 45-64, and ≥65 years), gender (female=1, male=0), education (less than high school, high school, some college, and college or greater), poverty level (poor: 0%-99% of the federal poverty level [FPL], near poor 100%-199% of the FPL, above poor: ≥200% of the FPL), marital status (married=1), employment status (employment=1), and living in a household with kids (children=1). We collapsed the first three categories of self-rated health (excellent, very good, good) into a single category (good) and compared that to all other responses. Additionally, we controlled for having a usual source of care other than the emergency department, being currently insured, and having been diagnosed with one of eight chronic diseases (asthma, diabetes or prediabetes, heart disease, stroke, arthritis, gout, or lupus).

Obesity was defined as having a body mass index (BMI) in the obese range according to Centers for Disease Control and Prevention (CDC) definitions. “Smoker” was defined as someone who has smoked at least 100 cigarettes in their lifetime and reported currently smoking some days or every day. Frequent binge drinking was defined as binge drinking (≥5 drinks for men and ≥4 drinks for women) monthly or more often. The majority of respondents reported eating less than seven servings of vegetables per week (respondents were asked how often they eat vegetables per week), so we dichotomized vegetable consumption into those who consumed vegetables six or fewer times per week and those who consumed vegetables seven or more times per week. We dichotomized variables representing unfavorable eating habits into consuming two or more servings of soda per week and consuming two or more servings of fast food per week. Additionally we dichotomized the sample into individuals who walked less than 150 minutes per week (for work or exercise).

### Other Measures

In models comparing Latinos to whites, we controlled for being Latino (Latino=yes, non-Hispanic white=no). As previously discussed, there exists significant demographic and cultural differences between US- and foreign-born Hispanics in the United States [23,24]. Therefore, we used the US Census definition of nativity, dividing Latino respondents into US- and foreign-born individuals. Because language use varies between US- and foreign-born Latinos and is related to Internet use among Latinos in the United States [9], we controlled for acculturation using English language proficiency and language in which media is consumed. We measured a respondent’s lack of acculturation via the proxies (1) respondent’s English proficiency is low and (2) respondent only consumes media in a non-English language.

We controlled for neighborhood-level factors with two social capital measures, trust/safety and civic engagement, constructed from individual-level social capital variables in the CHIS. Trust/safety measures respondents’ perceptions that people in their neighborhood can be trusted and are willing to help one another, their feelings of safety in their neighborhood, and perception that neighbors watch out for the safety of children in the neighborhood. Civic engagement measures volunteering in the community and attending meetings dealing with community problems during the past year. These measures were created by averaging the individual items in the CHIS following a factor analysis to ensure that all items loaded on a single factor.

### Analysis

We first conducted three logistic regressions comparing Latinos to whites to test if Latinos odds of (1) using the Internet, (2) engaging in online health information-seeking behavior, and (3) lack of confidence in filling out online forms were lower than non-Hispanic whites. Our sample population for this analysis were non-Hispanic whites and Latinos. We then used logistic regressions to (1) test whether US-born Latinos were more likely to use the Internet compared foreign-born Latinos, (2) examine the correlates of online health information-seeking behavior, and (3) examine the characteristics of Latino Internet users who were not confident filling out online forms. After we conducted the last two Latino analyses, we stratified our sample by nativity. All analyses were weighted according to CHIS directions.

## Results

In all, 6037 of 9506 (69.95%) Latinos reported using the Internet in the last year (Table 1). Of these 6037 Latinos, 3190 (53.36%) reported engaging in online health information-seeking behavior and 998 of 6037 (15.64%) reported a lack of confidence in filling out online forms.

**Table 1:** Descriptive characteristics of all Latinos and Latinos who use the Internet in the California Health Interview Survey (2011-2012).

Characteristics		Latinos (n=9506)	Latinos who use the Internet (n=6037)
Use the Internet, n (%)^a^		6037 (69.95)	—
Engage in online health information-seeking behavior, n (%)		—	3190 (53.36)
Lack online form confidence (not at all confident or not too confident filling out online forms), n (%)		—	998 (15.64)
**Nativity, n (%)**			
	Foreign-born	5425 (54.93)	2672 (42.33)
	US-born	4081 (45.07)	3365 (57.67)
**Acculturation, n (%)**			
	English proficiency is low	3659 (37.14)	1225 (20.52)
	Only consumes media in the non-English language	2482 (24.77)	720 (11.62)
**Age (years), n (%)**			
	18-24	1332 (18.70)	1296 (26.17)
	25-34	1474 (22.25)	1200 (26.16)
	35-44	1923 (22.58)	1338 (22.66)
	45-64	3354 (28.77)	1819 (22.30)
	≥65	1423 (7.70)	384 (2.72)
Female, n (%)		5531 (50.53)	3376 (49.97)
**Education, n (%)**			
	Below high school	3267 (34.98)	965 (18.71)
	High school	2676 (28.71)	2020 (33.92)
	Some college	2125 (22.13)	1753 (28.32)
	Bachelors or higher	1438 (14.17)	1299 (19.05)
**Poverty level, n (%)**			
	Poor (0%-99% of the FPL)	2939 (29.09)	1437 (23.05)
	Near poor (100%-199% of the FPL)	2768 (29.15)	1567 (26.87)
	Above poor (≥200% of the FPL)	3799 (41.76)	3033 (50.09)
Married, n (%)		4638 (46.92)	2802 (42.14)
Employed, n (%)		5890 (58.90)	3641 (64.10)
Lives in a family with children, n (%)		3881 (44.00)	2645 (44.03)
Self-rated health is good to excellent, n (%)		6540 (72.49)	4845 (81.46)
Has usual sources of care, n (%)		7553 (75.74)	4892 (77.11)
Currently insured, n (%)		7022 (70.05)	4485 (71.26)
Chronic diseases, n(%)		3276 (26.73)	1579 (20.18)
**Risk behavior, n (%)**			
	Obese	3240 (32.55)	1810 (28.73)
	Smoker	1014 (12.27)	661 (12.30)
	Binge drinks once a month or more often	886 (12.51)	742 (15.39)
	Drinks soda ≥2 times per week	3247 (39.55)	2185 (41.16)
	Eats ≥2 servings of fast food per week	3600 (44.34)	2708 (50.93)
	Eats <7 servings of vegetables per week	6012 (65.79)	3708 (64.54)
	Walks <150 minutes per week	8050 (84.14)	5146 (84.11)
**Social capital, mean (SD)**			
	Trust and safety (continuous)	2.94 (0.01)	2.94 (0.01)
	Civic engagement (continuous)	0.17 (0.003)	0.21 (0.004)

^a^ Percentages are weighted according to California Health Interview Survey directions in order to provide California population estimates.

### White Versus Latino Knowledge Gap

We found that Latinos were less likely to have ever used the Internet (OR 0.48, 95% CI 0.41-0.56, *P*<.001), to have engaged in online health information-seeking behavior (OR 0.71, 95% CI 0.63-0.80, *P*<.001), and were more likely to say that they were not at all or not too confident they could fill out an online application on their own (OR 1.7, 95% CI 1.36-2.12, *P*<.001) compared to non-Hispanic whites.

### Latino Internet Use

We found no significant differences in Internet use between US- and foreign-born Latinos when examining all Latinos (both US- and foreign-born, Table 2). Individuals with low English proficiency (OR 0.33, 95% CI 0.23-0.47, *P<*.001) or who consumed media in a non-English language (OR 0.46, 95% CI 0.35-0.60, *P*<.001) had lower odds of using the Internet. Individuals ages 25-34 (OR=0.19, 95% CI 0.12-0.31, *P*<.001) had lower odds than those aged 18 to 24 years; Latinos aged 65 or older (OR 0.01, 95% CI 0.01-0.02, *P*<.001) had significantly lower odds of using the Internet. Lower education and poverty levels were associated with never having used the Internet. Women (OR 1.28, 95% CI 1.04-1.59, *P=*.02), employed individuals (OR 1.29, 95% CI 1.04-1.60, *P=*.02), individuals living in a family with children (OR 1.37, 95% CI 1.07-1.75, *P=*.01), and with good self-rated health (OR 1.43, 95% CI 1.10-1.85, *P=*.008) had higher odds of ever using the Internet, whereas individuals with a chronic disease (OR 0.69, 95% CI 0.57-0.84, *P*<.001) had lower odds of ever using the Internet. When examining health-risk behaviors, individuals who were obese (OR 0.79, CI 0.64-0.98, *P=*.03) had lower odds of ever having used the Internet, whereas Latinos who were frequent binge drinkers (OR 1.49, 95% CI 1.07-2.08, *P=*.02), or who ate two or more servings of fast food (OR 1.65, 95% CI 1.31-2.07, *P*<.001) had higher odds of using the Internet. When examining social integration, individuals with higher levels of trust had lower odds of ever having using the Internet (OR 0.81, 95% CI 0.66-0.99, *P=*.04), whereas individuals who were civically engaged had higher odds (OR 3.13, 95% CI 2.18-4.50, *P=*.001) of ever having used the Internet.

### Online Health Information-Seeking Behavior

When examining online health information-seeking behavior among all Latinos (Table 3), we found that foreign-born Latinos (OR 0.71, 95% CI 0.58-0.88, *P*=.002) had lower odds of engaging in online health information-seeking behavior than US-born Latinos. Individuals aged 65 years or older (OR 0.54, 95% CI 0.40-0.75, *P*<.001), who had lower levels of education (below high school: OR 0.33, 95% CI 0.24-0.46, *P*<.001; high school: OR 0.53, 95% CI 0.41-0.67, *P*<.001; some college: OR 0.72, 95% CI 0.59-0.87, *P=*.001), and who were poor (OR 0.75, 95% CI 0.61-0.93, *P=*.01) had lower odds of engaging in online health information-seeking behavior. Women (OR 1.50, 95% CI 1.27-1.77, *P*<.001), individuals with a usual source of care (OR 1.43, 95% CI 1.14-1.79, *P*=.01), and individuals with a chronic disease (OR 1.26, 95% CI 1.02-1.57, *P=*.03) had higher odds of online health information-seeking behavior. When examining health-risk behaviors, individuals who smoked (OR 0.78, 95% CI 0.62-1.00, *P=*.047) had lower odds of health information-seeking behavior, whereas individuals who frequently binge drank had higher odds (OR 1.40, 95% CI 1.11-1.78, *P=*.006) of online health information-seeking behavior.

When stratifying by nativity (Table 4) we found that US-born Latinos who had a usual source of care (OR 1.56, 95% CI 1.17-2.08, *P=*.003) and binge drinking (OR 1.53, 95% CI 1.14-2.06, *P=*.006) had higher odds of online health information-seeking behavior, whereas these items were not associated with online health information-seeking behavior among foreign-born Latinos. Smokers had lower odds (OR 0.65, 95% CI 0.43-0.98, *P=*.03) of online health information-seeking behavior among foreign-born Latinos, but this was not associated with online health information-seeking behavior among US-born individuals.

**Table 2:** Logistic regression: correlates of Internet use among Latinos in California from CHIS 2011-2012 (N=9474).

Characteristics		OR (95% CI)	*P*
**Nativity**			
	Foreign-born	0.82 (0.60-1.12)	.21
**Acculturation**			
	English proficiency is low	0.33 (0.23-0.47)	<.001
	Only consumes media in non-English language	0.46 (0.35-0.60)	<.001
**Age (years)**			
	25-34	0.19 (0.12-0.31)	<.001
	35-44	0.13 (0.08-0.22)	<.001
	45-64	0.06 (0.04-0.10)	.001
	≥65	0.01 (0.01-0.02)	<.001
Female		1.28 (1.04-1.59)	.02
**Education**			
	Below high school	0.12 (0.08-0.20)	<.001
	High school	0.36 (0.23-0.56)	<.001
	Some college	0.51 (0.32-0.82)	.006
**Poverty level**			
	Poor (0%-99% of the FPL)	0.46 (0.35-0.61)	<.001
	Near poor (100%-199% of the FPL)	0.73 (0.57-0.95)	.02
Married		1.03 (0.81-1.32)	.81
Employed		1.29 (1.04-1.60)	.02
Lives in a family with children		1.37 (1.07-1.75)	.01
Self-rated health is good to excellent		1.43 (1.10-1.85)	.008
Has usual sources of care		1.20 (0.95-1.53)	.13
Currently insured		1.05 (0.81-1.37)	.68
Chronic diseases		0.69 (0.57-0.84)	<.001
**Risk behavior**			
	Obese	0.79 (0.64-0.98)	.03
	Smoker	0.82 (0.61-1.10)	.18
	Binge drinks once a month or more often	1.49 (1.07-2.08)	.02
	Drinks soda ≥2 times per week	1.09 (0.84-1.42)	.50
	Eats ≥2 servings of fast food per week	1.65 (1.31-2.07)	<.001
	Eats <7 servings of vegetables per week	0.92 (0.71-1.18)	.49
	Walks <150 minutes per week	1.04 (0.79-1.36)	.79
**Social capital**			
	Trust and safety	0.81 (0.66-0.99)	.04
	Civic engagement	3.13 (2.18-4.50)	<.001
	Constant	204.57 (86.81-482.07)	<.001

**Table 3:** Logistic regression: correlates of online health information-seeking behavior and lack of form confidence among Latinos in California from CHIS 2011-2012 (n=6035).

Characteristics		Online health information-seeking behavior		Lack of form confidence	
		OR (95% CI)	*P*	OR (95% CI)	*P*
**Nativity**				
	Foreign-born	0.71 (0.58-0.88)	.002	2.90 (2.07-4.06)	<.001
**Acculturation**				
	English proficiency is low	0.81 (0.61-1.07)	.14	2.51 (1.89-3.34)	<.001
	Only consumes media in non-English language	0.74 (0.54-1.03)	.08	1.46 (1.05-2.03)	.03
**Age (years)**				
	25-34	1.24 (0.94-1.62)	.12	0.87 (0.57-1.34)	.53
	35-44	1.04 (0.76-1.40)	.82	1.25 (0.79-1.99)	.33
	45-64	0.86 (0.66-1.13)	.28	1.10 (0.71-1.71)	.67
	≥65	0.54 (0.40-0.75)	<.001	2.03 (0.94-4.38)	.07
	Female	1.50 (1.27-1.77)	<.001	1.17 (0.90-1.51)	.23
**Education**				
	Below high school	0.33 (0.24-0.46)	<.001	2.67 (1.76-4.05)	<.001
	High school	0.53 (0.41-0.67)	<.001	1.89 (1.26-2.84)	.003
	Some college	0.72 (0.59-0.87)	.001	1.11 (0.71-1.73)	.63
**Poverty level**				
	Poor (0%-99% of the FPL)	0.75 (0.61-0.93)	.01	1.47 (0.98-2.22)	.06
	Near poor (100%-199% of the FPL)	0.91 (0.74-1.11)	.34	1.77 (1.28-2.45)	.001
Married		1.01 (0.82-1.25)	.92	1.23 (0.92-1.65)	.16
Employed		0.99 (0.83-1.18)	.92	0.96 (0.74-1.25)	.78
Lives in a family with children		0.95 (0.77-1.18)	.66	0.91 (0.67-1.25)	.56
Self-rated health is good to excellent		1.03 (0.82-1.29)	.81	0.62 (0.44-0.88)	.01
Has usual sources of care		1.43 (1.14-1.79)	.002	0.74 (0.52-1.04)	.08
Currently insured		1.06 (0.86-1.32)	.58	0.88 (0.61-1.26)	.47
Chronic diseases		1.26 (1.02-1.57)	.03	0.85 (0.60-1.21)	.37
**Risk behavior**				
	Obese	0.93 (0.76-1.12)	.44	0.99 (0.75-1.30)	.92
	Smoker	0.78 (0.62-1.00)	.047	1.15 (0.72-1.82)	.55
	Binge drinks once a month or more often	1.40 (1.11-1.78)	.006	0.78 (0.52-1.17)	.23
	Drinks soda ≥2 times per week	0.89 (0.74-1.07)	.21	1.12 (0.86-1.47)	.39
	Eats ≥2 servings of fast food per week	1.08 (0.91-1.29)	.39	0.72 (0.54-0.95)	.02
	Eats <7 servings of vegetables per week	0.88 (0.75-1.03)	.10	1.21 (0.93-1.57)	.15
	Walks <150 minutes per week	1.08 (0.86-1.37)	.49	1.02 (0.74-1.41)	.91
**Social capital**				
	Trust and safety	0.89 (0.77-1.04)	.13	0.70 (0.58-0.83)	<.001
	Civic engagement	2.64 (2.01-3.49)	<.001	0.69 (0.47-1.02)	.07
	Constant	1.74 (0.93-3.23)	.08	0.15 (0.06-0.37)	<.001

**Table 4:** Logistic regression: correlates of online health information-seeking behavior among Latinos in California stratified by nativity (source: CHIS 2011-2012).

Characteristics		US-born Latinos (n=3363)		Foreign-born Latinos (n=2672)	
		OR (95% CI)	*P*	OR (95% CI)	*P*
**Acculturation**				
	English proficiency is low	0.42 (0.17-1.00)	.049	0.78 (0.57-1.07)	.12
	Only consumes media in non-English language	0.70 (0.29-1.67)	.42	0.74 (0.52-1.05)	.09
**Age (years)**				
	25-34	1.08 (0.77-1.50)	.66	1.40 (0.89-2.20)	.14
	35-44	0.87 (0.54-1.40)	.55	1.21 (0.78-1.88)	.39
	45-64	0.80 (0.57-1.14)	.21	0.91 (0.58-1.44)	.69
	≥65	0.57 (0.37-0.89)	.01	0.47 (0.24-0.91)	.03
Female		1.41 (1.12-1.78)	.004	1.53 (1.17-1.99)	.002
**Education**				
	Below high school	0.33 (0.20-0.53)	<.001	0.36 (0.24-0.55)	<.001
	High school	0.46 (0.33-0.64)	<.001	0.64 (0.45-0.91)	.01
	Some college	0.66 (0.49-0.88)	.006	0.81 (0.57-1.14)	.22
**Poverty level**				
	Poor (0%-99% of the FPL)	0.77 (0.56-1.05)	.10	0.76 (0.53-1.09)	.13
	Near poor (100%-199% of the FPL)	0.94 (0.69-1.29)	.72	0.94 (0.68-1.31)	.73
Married		1.16 (0.89-1.52)	.26	0.90 (0.67-1.21)	.50
Employed		1.06 (0.86-1.31)	.58	0.87 (0.67-1.12)	.28
Lives in a family with children		1.01 (0.75-1.36)	.94	0.88 (0.67-1.16)	.35
Self-rated health is good to excellent		1.15 (0.82-1.62)	.40	0.92 (0.66-1.27)	.60
Has usual sources of care		1.56 (1.17-2.08)	.003	1.27 (0.92-1.76)	.15
Currently insured		1.09 (0.81-1.47)	.55	1.07 (0.79-1.46)	.67
Chronic diseases		1.28 (0.99-1.67)	.06	1.21 (0.88-1.65)	.24
**Risk behavior**					
	Obese	0.87 (0.67-1.13)	.29	1.02 (0.77-1.35)	.89
	Smoker	0.92 (0.67-1.25)	.58	0.65 (0.43-0.98)	.04
	Binge drinks once a month or more often	1.53 (1.14-2.06)	.01	1.16 (0.77-1.75)	.47
	Drinks soda ≥2 times per week	0.81 (0.64-1.02)	.07	1.01 (0.77-1.31)	.95
	Eats ≥2 servings of fast food per week	1.06 (0.83-1.35)	.63	1.10 (0.86-1.42)	.44
	Eats <7 servings of vegetables per week	0.94 (0.78-1.15)	.56	0.81 (0.62-1.05)	.10
	Walks <150 minutes per week	1.13 (0.83-1.54)	.44	1.05 (0.74-1.49)	.79
**Social capital**					
	Trust and safety	0.85 (0.69-1.06)	.15	0.93 (0.75-1.14)	.47
	Civic engagement	3.55 (2.34-5.38)	<.001	1.87 (1.20-2.91)	.01
	Constant	1.61 (0.71-3.61)	.25	1.45 (0.55-3.79)	.44

**Table 5:** Logistic regression: correlates of lack of online form confidence (not at all confident or not too confident filling out online forms) among Latinos in California stratified by nativity (source: CHIS 2011-2012).

Characteristics		US-born Latinos (n=3363)		Foreign-born Latinos (n=2672)	
		OR (95% CI)	*P*	OR (95% CI)	*P*
**Acculturation**					
	English proficiency is low	1.99 (0.74-5.3)	.17	2.56 (1.89-3.47)	<.001
	Only consumes media in the non-English language	1.82 (0.4-8.21)	.43	1.49 (1.10-2.02)	.01
**Age (years)**					
	25-34	0.57 (0.28-1.19)	.13	1.54 (0.85-2.79)	.16
	35-44	0.57 (0.22-1.45)	.23	2.14 (1.15-3.97)	.02
	45-64	0.89 (0.46-1.74)	.74	1.68 (0.95-2.97)	.07
	≥65	3.54 (1.40-8.96)	.008	1.39 (0.52-3.69)	.50
Female		1.30 (0.83-2.03)	.25	1.11 (0.79-1.56)	.53
**Education**					
	Below high school	9.35 (2.39-36.59)	.002	2.06 (1.34-3.16)	.001
	High school	5.17 (1.42-18.80)	.01	1.51 (0.95-2.42)	.08
	Some college	3.94 (1.10-14.11)	.04	0.81 (0.47-1.37)	.42
**Poverty level**					
	Poor (0%-99% of the FPL)	1.20 (0.64-2.23)	.57	1.57 (0.96-2.55)	.07
	Near poor (100%-199% of the FPL)	1.55 (0.91-2.66)	.11	1.73 (1.17-2.56)	.006
Married		1.09 (0.55-2.16)	.80	1.27 (0.93-1.74)	.16
Employed		0.68 (0.45-1.04)	.08	1.10 (0.78-1.54)	.58
Lives in a family with children		1.10 (0.51-2.35)	.81	0.81 (0.58-1.14)	.22
Self-rated health is good to excellent		0.41 (0.21-0.81)	.01	0.72 (0.50-1.04)	.08
Has usual sources of care		0.49 (0.24-1.00)	.049	0.87 (0.59-1.26)	.45
Currently insured		0.95 (0.49-1.84)	.88	0.84 (0.59-1.21)	.36
Chronic diseases		1.07 (0.57-2.02)	.83	0.82 (0.55-1.23)	.34
**Risk behavior**					
	Obese	0.80 (0.50-1.28)	.34	1.08 (0.77-1.50)	.66
	Smoker	1.44 (0.65-3.20)	.36	1.03 (0.64-1.65)	.90
	Binge drinks once a month or more often	0.60 (0.29-1.23)	.16	0.92 (0.56-1.51)	.75
	Drinks soda ≥2 times per week	0.88 (0.58-1.32)	.53	1.18 (0.87-1.60)	.28
	Eats ≥2 servings of fast food per week	1.06 (0.71-1.58)	.78	0.62 (0.44-0.87)	.01
	Eats <7 servings of vegetables per week	1.25 (0.73-2.15)	.40	1.19 (0.90-1.57)	.22
	Walks less than<150 minutes per week	1.18 (0.65-2.16)	.58	0.99 (0.67-1.45)	.95
**Social capital**					
	Trust and safety	0.78 (0.54-1.14)	.20	0.69 (0.55-0.87)	.002
	Civic engagement	0.44 (0.20-0.97)	.04	0.82 (0.52-1.29)	.39
	Constant	0.08 (0.01-0.58)	.01	0.29 (0.08-1.03)	.06

### Lack of Confidence in Filling Out Online Forms

Lack of confidence in filling out online forms was higher among Latinos with low English proficiency (Table 3, OR 2.51, 95% CI 1.89-3.34, *P*<.001) or those who only consumed media in a foreign language (OR 1.46, 95% CI 1.05-2.03, *P=*.03). Individuals with a high school or lower education level (below high school: OR 2.67, 95% CI 1.76-4.05, *P*<.001; high school: OR 1.89, 95% CI 1.26-2.84, *P=*.003) or who were near poor (OR 1.77, 1.28-2.45, *P=*.001) had higher odds of lacking confidence. Individuals with good self-rated health (OR 0.62, 95% CI 0.44-0.88, *P*=.01), who frequently ate fast food (OR 0.72, 95% CI 0.54-0.95, *P*=.03), or with higher levels of trust and safety were less likely to express a lack of confidence in filling out online forms.

When we stratified our sample by nativity (Table 5), we found that among US-born Latinos, elderly (≥65 years) individuals had higher odds (OR 3.54, 95% CI 1.40-8.96, *P=*.008) of expressing a lack of confidence. When examining socioeconomic status, we found that only education was related to lack of confidence. Although individuals with less than college had higher odds of expressing a lack of confidence filling out forms, Latinos with less than a high school education had more than 800% higher odds (OR 9.53, 95% CI 2.39-36.59, *P*=.002) of expressing a lack of confidence. Individuals with good self-rated health (OR 0.41, 95% CI 0.21-0.81, *P=*.01), who had a usual source of care (OR 0.49, 95% CI 0.24-1.00, *P=*.049), or who had higher levels of civic engagement (OR 0.44, 95% CI 0.20-0.97, *P=*.04) had lower odds of saying they were not confident filling out online forms. Among foreign-born Latinos, lack of English proficiency (OR 2.56, 95% CI 1.89-3.47, *P*=.001) and exclusive use of foreign language media (OR 1.49, 95% CI 1.10-2.02, *P=*.01) was related to lack of confidence filling out forms. Individuals who were near poor (OR 1.73, 95% CI 1.73-2.56, *P=*.006) or who lacked a high school education (OR 2.06, 95% CI 1.34-3.16, *P=*.001) had higher odds of not being confident filling out forms. Foreign-born Latinos who ate at least two servings of fast food per week or who had higher levels of perceptions of trust and safety in their neighborhood were less likely to express a lack of confidence with filling out online forms (OR 0.69, 95% CI 0.55-0.87, *P=*.002).

## Discussion

### Overview

Online interventions are viewed as a low-cost platform to deliver health information and interventions [38,39], but our findings show Latinos, particularly foreign-born Latinos, may not benefit from this shift to online delivery of health-related content and care. Our findings regarding a gap in Internet use and online health information-seeking behavior between non-Hispanic whites and Latinos are consistent with previous literature showing a disparity in Internet usage for online health information-seeking behavior in the general US population [15,18,19]. Our analysis adds to this literature in a number of ways. First, we show that there is a gap in reported confidence to fill out Internet forms between Latinos and non-Hispanic whites because Latinos have higher odds of not being confident in their ability to fill out online forms. Our study also contributes to the literature by showing a gap in online health information-seeking behavior and a confidence in filling out online forms between US- and foreign-born Latinos. The lower level of form-related confidence between Latinos and non-Hispanic whites may serve as a barrier to accessing health-related information through electronic records and in other important contexts. Improving confidence to fill out online forms may help in bridging this knowledge gap that Latinos have displayed in a number of contexts where filling out online forms allows them to participate in further information-seeking behavior. For example, many online smoking cessation and other forums require participants to provide health information and fill out forms. Feeling less confident or having difficulty with this type of task may limit access to these and other forms of online health information, such as medical records. Feeling less confident or having difficulty with online forms may also hinder the collection of health-related data on Latinos because they may be less inclined to participate in online data collection venues.

### Principal Findings

Education has been shown to be a social determinant of online health information-seeking behavior among the general US and the Latino population [26,40], but findings regarding age and gender have been mixed [11,15,26,27]. Our findings show that, consistent with studies on the general US population, education is related to online health information-seeking behavior [40]. Although one study found being male was related to online health information-seeking behavior among Latinos [26], our findings are consistent with other studies showing being female is associated with online health information-seeking behavior among the general US population, and Latinos in particular. We found that only those aged 65 or older had significantly lower odds of using the Internet compared to individuals aged 18 to 24 years. Studies of the US general population show individuals aged 65 or older are less likely to engage in online health information-seeking behavior [40]. A prior study showed language was a significant predictor of online health information-seeking behavior among Latinos [15], our findings show that this was a significant determinant among native-born individuals only. When considering confidence in filling out online forms, we found that low education was associated with lower levels of form-related confidence. Additionally, language was a significant predictor of confidence for foreign-born, but not US-born, Latinos.

Increasing digital literacy among Latinos in California (and elsewhere) should be a priority because these individuals are more likely to be never users or discontinued Internet users compared to non-Hispanic whites. They also have low levels of Internet efficacy, even when controlling for primarily speaking Spanish at home [19], although recent studies have shown that Internet access and online health information-seeking behavior among foreign-born Latinos may be increasing [28]. Our findings show that although males and those with lower education need to be targeted with education campaigns among the general Latino population [26], the correlates of online health information-seeking behavior varied by nativity. For example, a lack of English proficiency is significantly related to online health information-seeking behavior among US-born individuals, but related to lack of form confidence among foreign-born individuals. Previous studies have shown that in the general US population, those who have difficulty accessing care are more likely to access online health information-seeking behavior [41], but we found that US-born Latinos who did not have a usual source of care had lower odds of engaging in online health information-seeking behavior. There was no relationship between access to care and online health information-seeking behavior among foreign-born Latinos. Additionally, there appeared to be an association, although it was not statistically significant, with having a chronic disease and with online health information-seeking behavior among native-born individuals (*P=*.06), but not among foreign-born individuals (*P=*.24). Because chronic diseases require significant support, knowledge, and self-care, individuals with chronic diseases should be targets of campaigns to increase online health information-seeking behavior among foreign-born Latinos. When we examined individuals who engaged in health-risk behaviors, we found that only smoking and binge drinking were related to online health information-seeking behavior among all Latinos. However, we found that this association varied by nativity. Among native-born Latinos, binge drinking was associated with higher odds of online health information-seeking behavior, whereas among foreign-born individuals, smokers had lower odds of engaging in online health information-seeking behavior.

### Limitations

This study suffers from the limitations of cross-sectional self-reported data; causality cannot be determined and responses may be biased by the limitations of memory. Social desirability may also have biased responses, particularly when reporting characteristics such as weight and substance use. Additionally, general literacy, health literacy, computer literacy, and eHealth literacy—all of which influence online health information-seeking behavior—were not assessed by the CHIS. This sample is not a general US sample, but is limited to individuals in California. As a result, findings are not generalizable to the US population, but may suggest trends taking place in states with large Latino populations, particularly in the West and in areas with growing populations of Latinos.

### Conclusion

Latinos, particularly first foreign-born individuals, are at an increased risk of being left behind as the move to increase online content delivery and care expands. When considering Latinos, it is important to note that there are nativity differences in the correlates of online health information-seeking behavior. Our research also identifies a significant gap in confidence regarding filling out online forms between individuals who are first US- and foreign-born, whose English proficiency is low and who only consume media in non-English languages, particularly among foreign-born Latinos. This indicates that as health information and online health portals become more popular, education and training for foreign-born individuals, and online health portals that are in Spanish and use Spanish forms, should be considered. Additionally, education was significantly related to a lack of confidence in filling out online forms, indicating that perhaps usability and simplicity should be a priority for online sites. This may also indicate that until digital literacy can be increased among all groups, data may need to be gathered in offline formats.

There are some groups within our sample that engage in high-risk health-related behaviors. Those US-born Latinos who binge drink are less likely to search for health information-seeking behavior online, suggesting that alternate channels should be used to supply this population with health-related information. It may be possible to provide health messages in bars or other places where binge drinkers are likely to frequent. This has already been done in the context of tobacco control. Further, foreign-born Latinos who eat two or more servings of fast food per week were less likely to be confident in filling out online forms. This may suggest that in some communities in California, particularly those that contain a small number of fast food establishments that could be tracked by researchers, researchers could pilot an eHealth literacy intervention to take place in these establishments.

## Abbreviations

BMI: body mass index
CHIS: California Health Interview Survey
FPL: federal poverty level
